# On Elementary Affective Decisions: To Like Or Not to Like, That Is the Question

**DOI:** 10.3389/fpsyg.2016.01836

**Published:** 2016-11-24

**Authors:** Arthur Jacobs, Markus J. Hofmann, Annette Kinder

**Affiliations:** ^1^Department of Experimental and Neurocognitive Psychology, Freie Universität BerlinBerlin, Germany; ^2^Center for Cognitive Neuroscience, Freie Universität BerlinBerlin, Germany; ^3^Dahlem Institute for Neuroimaging of Emotion, Freie Universität BerlinBerlin, Germany; ^4^Department of General and Biological Psychology, Bergische Universität WuppertalWuppertal, Germany; ^5^Department of Education and Psychology, Freie Universität BerlinBerlin, Germany

**Keywords:** neurocognitive poetics, elementary affective decisions, liking, beauty, neuroaesthetics, ludic reading, decision tree modeling, basic affective tone

## Abstract

Perhaps the most ubiquitous and basic affective decision of daily life is deciding whether we like or dislike something/somebody, or, in terms of psychological emotion theories, whether the object/subject has positive or negative *valence*. Indeed, people constantly make such liking decisions within a glimpse and, importantly, often without expecting any obvious benefit or knowing the exact reasons for their judgment. In this paper, we review research on such *elementary affective decisions (EADs*) that entail no direct overt reward with a special focus on *Neurocognitive Poetics* and discuss methods and models for investigating the neuronal and cognitive-affective bases of EADs to verbal materials with differing degrees of complexity. In line with evolutionary and appraisal theories of (aesthetic) emotions and data from recent neurocognitive studies, the results of a decision tree modeling approach simulating EADs to single words suggest that a main driving force behind EADs is the extent to which such high-dimensional stimuli are associated with the “basic” emotions joy/happiness and disgust.

“How then did it work out, all this? How did one judge people, think of them? How did one add up this and that and conclude that it is liking one felt, or disliking?”

*– Virginia Woolf*, *To the Lighthouse*

## Introduction

Deciding whether we like or dislike something/somebody, i.e., – in terms of psychological emotion theories – whether it has positive or negative *valence* is perhaps the most ubiquitous and basic affective decision of daily life. We constantly make such *liking decisions* within a glimpse – not only in Facebook –, and often without expecting any obvious benefit or knowing the exact reasons for our judgment. Here we would like to introduce the term *elementary affective decisions (EADs*) for this kind of ubiquitous decisions that entail no direct overt reward. While EADs are made with regard to all types of perceived objects in daily life (Lebrecht et al., 2012), here we focus on EADs concerning verbal materials in the context of literature reception and reading from a *Neurocognitive Poetics* perspective, i.e., the investigation of the neuronal, experiential, and behavioral effects produced by literary texts (Jacobs, 2015b). This has several advantages. First, there exist well validated databases allowing to quantify not only the valence of written words and texts, but also various other features that may interact with valence in the process of EADs, e.g., stimulus arousal or imageability. Second, the majority of empirical studies on EADs has used words as stimuli thus providing a solid basis for review and model development (e.g., Maddock et al., 2003; Võ et al., 2006; Unkelbach et al., 2008; Jacobs et al., 2015; Sylvester et al., 2016). Finally, the focus on words having quantifiable features allows computational modeling of EADs testing the main hypotheses proposed in this paper.

The central working hypothesis we pursue in this paper is that the computed valence of single words and the mostly preconscious and preverbal EADs accompanying word recognition forms the basis of pleasurable aesthetic literary experiences with more complex verbal materials. A more specific hypothesis states that valence is a *semantic superfeature* that results from a yet unknown integration of both experiential and distributional data, at least partially represented in associative activation patterns of semantic networks, as assumed by the semantics theory of Andrews et al. (2009). A valence value would thus be computed from (1) neural activation patterns distributed over the sensory-motor representations of a word’s referents (*experiential* aspect) and (2) the linguistic company the words keep (Harris, 1951), i.e., the size and density of their context (*distributional* aspect; Jacobs et al., 2015), as computationally modeled using co-occurrence statistics (Hofmann and Jacobs, 2014). In favor of this view, Westbury et al. (2014) recently showed that valence ratings of words can be predicted by their associations to a selected set of emotion labels, derived from theories of basic emotions (cf. also Hofmann and Jacobs, 2014). More recent behavioral and neurocognitive evidence stemming from experiments using words of neutral valence and bivalent noun-noun compounds (i.e., novel words created by fusing two nouns that have different valences, such as BOMBSEX) supports this view (Kuhlmann et al., 2016).

In the following we start with a short review of theories of emotion, aesthetics, and reading relevant for our purposes. A section on “Methods and Materials” used in the study of EADs then prepares the next section which introduces factors determining the liking and neuronal correlates of verbal materials with increasing degrees of complexity. A final section presents formal models of EADs with different degrees of complexity.

## Liking Words and Ludic Reading

Reading is indeed one of those ubiquitous activities often chosen for pleasure, and the mental and neuronal processes underlying ludic reading and literary experience in general have been the object of increasing research efforts (e.g., Nell, 1988; Anz, 1998; Schrott and Jacobs, 2011; Jacobs, 2015c; Jacobs and Schrott, 2015). Although little is known about the processes underlying children’s learning to like things, in particular regarding pleasurable literary experiences, there are impressive testimonies by children explaining, for example, why they find even single words beautiful (Limbach, 2004; Jacobs et al., 2015): A phenomenon that could be termed *episodes of micropoetry* (cf., Jacobs, 2015c; Jacobs and Kinder, 2015). Learning to like language is a life-long process starting from the earliest days, e.g., through discovering new meanings offering the promise of novel insights, enjoying word plays, sounds and rhythms, self-rewarding feelings of suspense, surprise and vicarious joy, fear or disgust. As argued elsewhere, later joyful or enlightening encounters with poetry are likely to be rooted in early life experiences with micropoetry. These include preverbal and preliterate *(micro)poetic episodes*, e.g., lullabies, nursery rhymes, and word games, making children enjoy rhyme and delight in rhythm and repetition (Freud, 1907; Jakobson, 1944; Miall and Dissanayake, 2003; Wainwright, 2011; Jacobs, 2015c; Jacobs and Kinder, 2015).

It also has been argued that from early, preliterate childhood on, word games play an important role in all kinds of incidental or ludic learning activities, as well as in emotion regulation and mood management and that these activities are the precursors of ludic reading and aesthetic liking accompanying many subsequent literary experiences (Jakobson, 1944; Jacobs, 2015c; Jacobs et al., 2015; Pompe, 2015). Burke (2013, p. 208) wonderfully expresses this: “when he/she subconsciously longs for the style figural, rhythmic rewards….that sense of balance, of perfection, of exactitude (in the context of adults reading Joyce’s short story “The dead”), or “how strong the subconscious desire is to be moved by emotive literary style fragments.” But this raises the question of how children and young adults acquire these literary longings and desires. With regard to children reading Dr. Seuss’ *Horton Hears a Who*, Boyd (2001, p. 204) puts it this way: “He (i.e., the author) can count on children’s pleasure that someone has made up a story for them to respond to, as a parcel of pleasure, a gift of attention.”

Within the field of neurocognitive poetics (Jacobs, 2015a, 2016b; see also Burke, 2015) we are only beginning to understand the potential causes and effects of ludic reading. One line of theorizing trying to explain what motivates readers to pass time with written stories or poems highlights the role of *immersion*. The multidimensional construct of immersion, as first proposed by the film theoretician Bela Balázs, and then applied to literature reception by Schrott and Jacobs (2011), is perhaps best described as *getting lost in a book* (Nell, 1988). Other metaphors than Balázs’ are popular in the literature, e.g., absorption or transportation, but at present both the conceptual work and empirical data base is not enough developed to allow sharp distinctions (Jacobs and Schrott, 2015; Jacobs and Lüdtke, in press). What seems clear is that young and adult readers long for immersion into novels like *Harry Potter* and a few pioneering studies have begun to investigate the neurocognitive and –affective underpinnings of this phenomenon (Hsu et al., 2014; Lüdtke et al., 2014). It is less clear whether readers’ longing for poetry like Shakespeare’s or Pushkin’s, as well as highly foregrounded narratives from Joyce or Proust is based on the same kind of neuropsychological processes or linked more to processes of aesthetic appreciation, as proposed by the Neurocognitive Poetics Model (NCPM; Jacobs, 2011, 2015a, 2016b; cf. also Miall, 1989; Lüdtke et al., 2014; Nicklas and Jacobs, 2016; cf. also Van den Hoven et al., 2016; Willems and Jacobs, 2016).

Although investigating the liking of complex and extensive verbal materials like novels is a worthwhile scientific activity (Burke, 2011), empirical studies on the possible foundations of pleasurable literary experiences, e.g., studying EADs with single words or sentences, remain a valuable source of insight, as do theoretical analyses in the light of more general theories on emotion or aesthetics. In the following we briefly discuss some relevant literature.

## The Role of Valence and Beauty in Theories of Emotion, Aesthetics, and Reading

Historically, psychology has investigated EADs or liking judgments from two major perspectives: emotion theories (e.g., Wundt, 1896) and theories of aesthetic judgment or beauty evaluation (e.g., Eysenck, 1941; Berlyne, 1971; Leder et al., 2004; Jacobsen, 2006; Nadal, 2013; Chatterjee and Vartanian, 2014; Leder and Nadal, 2014). Both perspectives produced a wealth of models and methods utilizing countless stimuli and tasks, but have been pursued relatively independently of each other without much theoretical and methodological cross-over (but see Pelowski et al., 2016). As a consequence, the issue whether liking judgments tap into the same underlying processes when participants are, for example, asked to rate the valence of emotion inducing pictures or words (i.e., in emotion-related research) as compared to when they are invited to rate the beauty of paintings, music, proverbs, or poems in research on (neuro-)aesthetics, still is an open one (cf. Marin, 2015; Omigie, 2015). Finding something/someone pleasurable and finding something/someone beautiful are logically independent, e.g., when liking something/someone because of other aspects than aesthetic ones, or attributing aesthetic value to a piece of art without finding it beautiful (i.e., without producing an experience of aesthetic pleasure). However, *psycho*logically pleasure and beauty seem intertwined, e.g., in appraisal theories (e.g., Silvia, 2009) or semantic differentials (Berlyne, 1971) they are often indistinguishable (i.e., hedonic terms). Empirically one also often finds a high correlation between liking and beauty judgements (e.g., Lüdtke et al., 2014). This fits with the classical notion shared by scholars as different as Kant, Gadamer, or Ramachandran that pleasure (associated with valence) is a necessary component of aesthetic feelings (Brown et al., 2011; Jacobs, 2015b).

A central goal of neuroaesthetics thus consists in *naturalizing* aesthetics by grounding aesthetic feelings in general theories of emotion (Brown et al., 2011). The latter authors argue that aesthetic processing is, at its core, the appraisal of the valence of perceived objects^1^. This (or a similar type of) appraisal is not limited to artworks (including verbal ones) at all, but is applicable to all types of perceived objects in daily life (Lebrecht et al., 2012). A recent meta-analysis on the topic (Kühn and Gallinat, 2012) concludes that subjective EADs are directly related to brain regions that have been described as part of the reward circuitry (e.g., medial orbitofrontal cortex, ventral striatum), and that the evaluation of likability is an automatic process that it is neither elicited nor enhanced by instructions to report the outcome of these judgements. In sum, human neuroimaging studies suggest that brain areas associated with aesthetic responses to artworks overlap with those that are linked to the appraisal of objects of evolutionary importance (such as desire for food or attractiveness of faces).

A plausible working hypothesis thus is to assume that artworks have co-opted the neural systems that subserve these kinds of adaptive assessments rather than having evolved a distinct type of neural processing (Brown et al., 2011). In the emerging field of neurocognitive poetics, not addressed in Brown et al.’s meta-analysis, the Panksepp-Jakobson Hypothesis (Jacobs, 2015b) expresses this similarly in an attempt to bridge the language-emotion gap. This concerns the gap between neurobiological theories of emotion, as perhaps best represented by Panksepp’s (1998) core affect systems theory, and complex (psycho-)linguistic models, as exemplified by Jakobson’s (1960) extended version of Bühler’s (1934) ‘organon model’ of language functions (Jacobs et al., 2015; see Koelsch et al., 2015, for a theory linking emotion and language). Stated simply, the Panksepp-Jakobson Hypothesis submits that since evolution had no time to invent a proper affective system for art reception, even less so for reading, the emotional and aesthetic processes we experience when reading must be somehow linked to the ancient neuronal affect circuits we share with all mammals (Panksepp, 1998). There is accumulating evidence for this hypothesis stemming from work on neurocognitive poetics generalizing across various stimulus materials, subjects, and tasks (for review, see Jacobs, 2015b).

In sum, it seems safe to say that liking, i.e., experiencing pleasure in terms of emotion theories – be it with positive or negative valence, or a mixture of both –, plays a role in feelings of beauty, most certainly when the latter are compressed into numerical ratings. To say it in the words of Silvia (2009, p. 48): “The psychology of aesthetic experience is eerily close to the psychology of how much novices say they like something.” However, the heterogeneity of notions, e.g., aesthetic experience, aesthetic episode, aesthetic appraisal, aesthetic judgment, aesthetic feeling/emotion, aesthetic evaluation etc., methods and materials used to assess felt beauty make it difficult to draw more precise conclusions at this stage (cf. Güçlütürk et al., 2016; Marin et al., 2016; Pelowski et al., 2016). In the following sections, we examine methods used to measure EADs in the context of verbal materials of differing degrees of complexity.

## Methods and Materials for Measuring Eads

The simplest task used to measure EADs is the valence decision task (VDT^2^) asking participants to explicitly decide whether a stimulus is positive or negative (pleasant/unpleasant; beautiful/ugly), either while recording their response times (RTs) or ratings, or both (e.g., Hill and Kemp-Wheeler, 1989; Maddock et al., 2003; Kawabata and Zeki, 2004; Võ et al., 2006, Võ et al., 2009; Unkelbach et al., 2008; Jacobs et al., 2015; McQuire et al., 2016; Sylvester et al., 2016). In the more complex valence rating task (VRT) participants typically are asked to rate the valence of stimuli on a scale (from -3 to 3, or 1-X, where X can be 5, 7, or even greater numbers). Both the VDT and VRT have produced ubiquitous phenomena with various types of stimuli (e.g., pictures, words, faces, paintings): (1) the inversely, slightly asymmetric U-shaped function relating RTs in the VDT to stimulus valence (e.g., Võ et al., 2006) or beauty (Kawabata and Zeki, 2004), and (2) the asymmetric U-shaped function relating valence to arousal ratings in the VRT (e.g., Võ et al., 2009; Schmidtke et al., 2014). We have discussed theoretical reasons for these phenomena elsewhere (Sylvester et al., 2016; cf. Ashby and Isen, 1999; Koch et al., 2016) and will not go into details here. For the present purposes, however, two points are important: first, negative stimuli are usually more arousing than positive ones, and, second, positive ones typically are processed faster. One lesson to learn for studies of EADs then is that the factor arousal must not be neglected or ignored, as is often the case in studies of reading. The second, more tricky issue concerns the dimensionality of the construct valence.

Wundt’s (1896) original conception of valence as a *bipolar* dimension had an important methodological (or measurement-theoretic) implication: valence typically is rated on *bipolar* scales (i.e., from very negative to very positive or very pleasant to very unpleasant). Thus, when a word or poem is judged as more pleasant, it automatically becomes less unpleasant. However, this notion has been challenged by theories that conceive valence as a *bivariate* construct which would require a different way of measuring it (e.g., Norris et al., 2010; Briesemeister et al., 2012). The issue is crucial because if a stimulus such as a word, face, or object can have positive *and* negative valence at the same time, i.e., is affectively ambivalent, this makes a huge difference especially with respect to aesthetic theory which has often highlighted the role of mixed emotions for aesthetic liking. For verbal materials, there is some preliminary evidence in favor of the *bivariate* perspective on valence coming from a study on words taken from the “Berlin Affective Word List” (BAWL; Võ et al., 2006, Võ et al., 2009). However, Briesemeister et al’s. (2012) study seems to be the only one addressing this issue with word materials, and thus the literature remains inconclusive resulting in several competing (descriptive) models of the VDT (Jacobs et al., 2015).

The second big issue concerns factors determining the valence of a stimulus and driving an EAD. What exactly is it that, for instance, makes people judge a word as pleasant or unpleasant, beautiful or ugly? Words are quasi-ideal stimuli to tackle this issue, because more than 50 quantifiable word features are known (Graf et al., 2005) that can be examined with regard to their role in valence judgments (Jacobs et al., 2015). As outlined in the “Introduction,” the latter authors recently proposed word valence to be a *semantic superfeature* resulting from a combination of *experiential* and *distributional* aspects. They identified 14 variables that contribute to this *mixed semantic structure* and affect EADs to words: three affective-semantic variables (valence, arousal, imageability), three (sub-)lexical ones (word frequency, number of syllables, neighborhood density), five discrete emotion variables (joy/happiness, fear, anger, sadness, disgust; Briesemeister et al., 2011, 2014, 2015), and three embodiment ones (taste, grasp, move). Among the discrete emotions, joy and disgust may play a special role for liking decisions since from a Darwinian perspective they form a logical pair, two sides of a medal, related to incorporation of attractive food (or mating partners) on the one hand (joy) and rejection of aversive food on the other (disgust). These sociobiological functions of incorporation and rejection can be generalized to symbolic stimuli thus forming a potential evolutionary, discrete emotion-related basis of liking and disliking (see section on modeling at the end of this paper). Similarly, joy and disgust also play a role in appraisal theories of aesthetic emotions (e.g., Silvia, 2009; cf. also Pelowski et al., 2016).

Altogether, the above considerations suggest that *To Like Or Not To Like* a single word is already a complicated multidimensional affair relativizing the above discussion about the dimensionality of the valence construct. So how can we explain EADs to multiword artifacts and verbal artworks such as metaphors, proverbs, idioms, sentences, passages, or entire stories and poems (Jacobs, 2016b)?

## Factors Influencing and Neuronal Correlates of Eads to Verbal Materials

The next section reviews features of words and text segments that have been shown to affect EADs to verbal materials. These features *per se* obviously provide neither a necessary nor a sufficient criterion for a piece of text to be judged as beautiful or poetic, given the intricate dynamics of the text-reader-context nexus (Jacobs, 2015c, 2016a) and the hierarchical and dynamic nature of beauty judgments (Kintsch, 2012).

## Single and Compound Words

When participants quickly (i.e., automatically) *decide* whether they like a word or not, as in the VDT, a complex of more than 10 variables accounts for variance in RTs (see above). To give an illustrative example from research with children (8–12 years old), the five most liked words in the kidBAWL database (Sylvester et al., 2016) were: NATURE, MAMA, GIFT, SMILE, and FRIEND; those least liked were: VIOLENCE, MURDER, CADAVER, DECEPTION, and STEAL. As concerns the example MAMA, the phylo- and ontogenetically determined optimal combination between *euphony* and *eusemy* can explain its automatically perceived beauty (Jacobs, 2015c), but when participants *rate* the beauty/ugliness of words in a less automatic, more time-consuming procedure, it is likely that even more (and other) factors enter into the mental equation. Regrettably, empirical work on the beauty of words is almost absent, but some preliminary data from a study on 450 words chosen from databases like “The most beautiful German Word” (Limbach, 2004), dictionaries of German adolescent language, and the BAWL, suggest that reference to phenomena from nature (e.g., animals, flowers, rainbow etc.) and states/objects of wellness (e.g., coziness), together with high (positive) valence, familiarity, and imageability values, and low arousal values, make words appear more beautiful^3^. The most beautiful words in the sample were LIBELLE (dragonfly), MORGENRÖTE (aurora), and MITTSOMMERNACHT (midsummernight). In contrast, the ugliest words in that sample were almost all swear words associated with genitalia (Jacobs et al., 2015: Supplementary Materials).

As more fully discussed in Jacobs et al. (2015), the 9-year old Sylwan Wiese explains why the word LIBELLE (dragonfly) is the most beautiful for him by refering to three cues: a perceptual one (the wobbling, a motion which he loves watching), a phonological one (the Ls which make the word glide so well on his tongue), and an affective-semantic feature (no fear, because the word itself expresses the feeling evoked by the wobbling ensuring that one is not afraid of these insects). Thus, for this child and perhaps also for others it seems that both associations with discrete emotions and embodied cognitions play a role in aesthetic appreciations of words. In terms of Kintsch’s (2012) computational model of beauty, words like MAMA or LIBELLE may be likeable and beautiful because the divergence between the distinctive codes for the perceptual-imageable, (sublexical) phonological and affective-semantic *parts* and the (lexico-semantic) code for the word as a whole is small, thus giving it a good Gestalt.

In one of the first neuroimaging studies examining word valence effects in an implicit task not requiring any attention to affective or aesthetic word properties (lexical decision), Kuchinke et al. (2005) found selective activations for positive words in brain regions associated with reward and semantic retrieval (orbitofrontal cortex; middle temporal gyrus). This is in line with the above mentioned neuroaesthetic studies and, moreover, with the idea that *liking words is related to a more fluent retrieval from semantic memory* (i.e., their familiarity). If EADs are (also) a function of the perceiver’s processing dynamics (Reber et al., 2004) – i.e., the more fluently readers can process a word or text segment, the more positive is their aesthetic liking response – then the more elaborated and interconnected (cohesive) schemata of positive words or objects (Ashby and Isen, 1999; Sylvester et al., 2016) may be a key factor explaining liking. Recent neurocognitive and -computational evidence supports this account by showing that pleasant words take advantage of a primarily left amygdala-mediated enhanced perceptual processing (Herbert et al., 2009) and that they provide more and denser semantic long-term associations than neutral or negative words (Hofmann and Jacobs, 2014). The link between memory associations and liking (positive valence) can be further explained in terms of complementary learning systems theory (Kumaran and McClelland, 2012), and by the hypothesis that the inferential function of the hippocampus can create a lucid positive feeling that may be figuratively described by the ‘light of understanding’ (Hofmann and Kuchinke, 2015). Thus, the neurocognitive function of recurrent feedback from the hippocampus to the temporal cortex can be circumscribed by Oscar Wilde’s adage “anything is good that stimulates thought” (Hyde, 1962, p. 108).

There are also studies providing data on the liking of more complex words, e.g., noun-noun compounds (NNCs) which are a very productive word class in German: One can create countless neologisms by coupling noun pairs (or, to a lesser extent, adjective/verb-noun pairs) varying along several theoretical dimensions such as familiarity, literality-metaphoricity (Forgács et al., 2012), or valence (Jacobs et al., 2015; Kuhlmann et al., 2016). With regard to the issue at hand, NNCs are indeed very interesting, because they allow to couple nouns of opposite valence, thus creating *bivalent words* that challenge EADs by creating a decision conflict. Jacobs et al. (2015) presented preliminary evidence that such newly created bivalent NNCs (e.g., bombsex) prolonged RTs in a VDT as compared to univalent NNCs (e.g., pimple-horror), likely due to the decision conflict interfering with meaning construction. The most liked NNCs in Jacobs et al.’s corpus were the neologisms: “Glücksgenuss” (happiness-enjoyment), “Fabelliebe” (fable-love), and “Traumfreiheit” (dream-freedom), the least liked were: “Gewaltgeschwür” (violence-abscess), “Foltervorwurf” (torture-reproach), and “Schurkentyrann” (rascal-tyrant). Neurocognitive experiments examining the processing of NNCs like “DUFTGESANG” (fragrance-chant; Kuhlmann et al., 2016) suggest that they require additional semantic integration work, as correlated with increased left inferior frontal gyrus activity as a function of both their familiarity and figurativeness (Forgács et al., 2012). To what extent the figurativeness or metaphoricity of NNCs or other words contributes to their liking has not yet been investigated, but is an interesting issue for future studies (cf. section on idioms below).

More generally concerning neural correlates of word valence, *positivity* is neuroanatomically most often associated with the basal ganglia including the ventral striatum, left frontal pole, medial orbitofrontal cortex, ventromedial prefrontal cortex, posterior cingulate cortex, and supplementary motor area, whereas *negativity* is rather associated with insula, right amygdala, periaqueductal gray, right dorsal anterior cingulate cortex, left orbitofrontal cortex, dorsomedial prefrontal cortex, and deep cerebellar areas. Amygdala and anterior insula activations – or an amygdalar-hippocampal network – are specifically associated with word arousal (see Citron, 2012, and Jacobs et al., 2015, for reviews). The anterior insula seems to play a key role in *disliking* words. Ponz et al. (2013) used ugly or disgusting words taken from the BAWL to test whether brain regions involved in processing emotional information in general (e.g., in faces, pictures, or smells) are also in charge of the processing of emotional information in words. Comparing the processing of words like VIRUS, AMPUTATION, or PISS to that of control stimuli, they found support for this specific variant of the Panksepp-Jakobson Hypothesis. In sum, simplifying an ever increasing heterogeneous complex of neurocognitive results, one could propose that EADs to words are primarily associated with orbitofrontal networks including the dorsolateral prefrontal cortex (Herrington et al., 2005) and the insular cortex.

## Multiword Expressions

Multiword expressions that form idioms or proverbs (see Cacciari, 2014, for review) have been examined with regard to their likeability and beauty in both behavioral and neurocognitive studies (Bohrn et al., 2012a,b, 2013; Citron and Goldberg, 2014; Citron et al., 2015, in revision).

### Idioms

Idioms are word strings whose global meaning cannot generally be inferred merely on the basis of the meaning of the constituent words (i.e., semantic transparency), and therefore has to be retrieved from semantic memory (Cacciari, 2014). Idioms like “Pfeffer im Hintern haben” (to have pepper in the ass) thus differ from proverbs, which, as literally and figuratively true statements, usually are temporarily undefined full sentences, signaled by specific grammatical, phonetic, and/or rhetorical patterns, or by a binary structure (theme/comment). Although some idioms can diachronically come from metaphors, they also differ from them, since metaphors (even the most frozen/dead ones) do not possess a unique standardized meaning and can convey more than one meaning depending on context (Citron et al., 2015). The most liked idioms in the German database of over 600 stimuli by Citron et al. were: “vor Freude strahlen” (to beam of joy), “im siebten Himmel sein” (to be in seventh heaven), and “auf Wolke sieben schweben” (to float on cloud seven).

Besides reproducing the above-mentioned asymmetric U-shaped function relating valence to arousal ratings observed with words (suggesting that idioms are affectively semantically processed like single words), the data of Citron et al. also allow to examine to what extent (rated) figurativeness contributes to the liking (rated valence) of multiword expressions. To illustrate contrasting degrees of figurativeness of German idioms consider the following examples for low figurativeness: “Das ist keine Kunst” (literal translation: this is no art; meaning: this is not difficult), or “keinen Pfennig mehr haben” (literal translation: to have no more cent; meaning: to be broke/out of money). In contrast, idioms exposing high figurativeness ratings are: “einen Kater haben” (literal translation: to have a tomcat; meaning: to be hung-over), or “grün hinter den Ohren sein” (literal translation: to be green behind the ears; meaning: to be immature/unexperienced). The results in Table 8 of Citron et al. (2015) suggest that EADs to idioms depend on their *familiarity*, *arousal* values, and *figurativeness*. Since figurativeness also correlated negatively with both concreteness and semantic transparency ratings (and positively with idiom length, i.e., log number of words/idiom), the data of this study tentatively suggest that *idioms are liked more the better they are known and understood* (i.e., familiarity), very likely reflecting the ubiquitous relation between processing fluency and aesthetic pleasure discussed above.

Citron et al. (in revision) recently compared idiomatic and literal sentences of negative, neutral, and positive valence in an fMRI study. They found that idioms elicited significantly enhanced activation of the left amygdala, and bilateral inferior frontal gyrus, the right temporal cortex, and the right precentral gyrus. Valence effects were seen in brain areas associated with language comprehension and conceptual representations, i.e., the left pre- and post-central gyri and the right superior temporal gyrus, whose activity was enhanced by both positively and negatively valenced stimuli, but not in the typical “emotion-related” areas discussed above.

### Proverbs

According to some authors, the *little pearls of wisdom* called proverbs have a special integrative potential reaching into the structure of human awareness where cognition, emotion, and volition often lose touch with each other to the detriment of all three (Hernadi and Steen, 1999). Their poetic and often anarchic offspring, so-called *anti-proverbs*, represents a special case of linguistic adaptation – more or less artful alternations of original proverbs like *A Rolling Stone Gathers Momentum* (Mieder, 2004; Jacobs, 2015c; Nicklas and Jacobs, 2016).

In Bohrn et al.’s (2012b, 2013) neurocognitive studies comparing familiar vs. unfamiliar proverbs with anti-proverbs, the most beautiful proverbs were familiar ones like “Wissen ist Macht” (knowledge is power) or “Wer wagt, gewinnt” (who dares, wins) thus outperforming the witty, artfully twisted anti-proverbs through the powers of familarity and processing fluency (Reber et al., 2004). In terms of Kintsch’s (2012) model of beauty, proverbs might represent a verbal construction that minimizes complexity by averaging and compressing a (deeper) meaning over many instances of utterances/sentences that may not be quite *prägnant* or beautiful in themselves, thus ironing out the imperfections of particular instances and yielding an idealized verbal image. Anti-proverbs like “Mens sana in campari soda” (Nicklas and Jacobs, 2016) are interesting stimuli because they present a nice example of how background and foreground features can be combined in a single sentence (Jacobs, 2015b): Due to their multiple rhetoric features (phonological similarities like rhyme/alliteration, repetition and parallelism, meter, brevitas, i.e., artful shortness/prägnanz, or ellipses), all proverbs can be considered foreground elements of language if seen against a background of literal, i.e., non-rhetorical, non-figurative control sentences. However, while the memory of the original proverb (e.g., ‘All roads lead to Rome’) provides familiar background information, the one-word change in anti-proverbs like ‘All sins lead to Rome,’ creates a foregrounding effect and a subjectively felt tension, perhaps similar to puns. It also produces affective and aesthetic responses, the neuronal traces of which can be measured using fMRI. Thus, Bohrn et al. (2012b) observed data compatible with the interpretation that anti-proverbs evoking two contrasting responses that have to be related (that of the familiar proverb and the novel word), required a greater semantic integration effort, as mirrored by stronger ventral inferior frontal gyrus activation than for control stimuli (much as with the aforementioned bivalent NNCs; Forgács et al., 2012; Kuhlmann et al., 2016). Moreover, concurrent increased medial orbitofrontal cortex and striatal activation likely reflected the rewarding aspect of successful semantic integration (a kind of “Aha” experience; Bar et al., 2006; Hofmann et al., 2014) and supports both the findings and views of the *naturalizing aesthetics* studies discussed above.

In sum, various stimulus features and neuronal networks are involved in EADs to multiword expressions. At the experiential and behavioral levels of observation, the superfeature valence together with arousal, familiarity/fluency, and minimal complexity appear to be key factors. At the neuronal level, apart from the reward circuitry networks associated with emotional engagement, semantic integration, and language processing in general play a role (Bohrn et al., 2012a).

### Sentences

“A nice line” is an often-used expression for phrases or sentences that, like the political slogan “I like Ike,” apply the two major principles of the poetic genre, i.e., the prominence of sound properties and more or less subtly expressed or perceived affective meanings, in an affectively and aesthetically appealing way (Jakobson, 1960; Schrott and Jacobs, 2011; Aryani et al., 2016). “What are men to rocks and mountains?” (Jane Austen, *Pride and Prejudice)* or “At the still point, there the dance is” (T. S. Eliot, “Four Quartets”) are examples for *nice lines* from novels and poetry. Fully explaining what makes such lines likeable or beautiful is beyond the powers of this paper. However, given our initial hypothesis that the computed valence of single words – and the mostly preconscious and preverbal EADs accompanying single word recognition – forms the basis of pleasurable, aesthetic literary experiences with more complex verbal materials, we can try to answer the simpler question: To what extent do the factors known to determine EADs to single words also co-determine the liking of sentences.

The simplest model predicting the liking of a phrase or sentence should take into account only the affective meaning of its component words while neglecting other potentially relevant influences like word type, order, or syntactic role, or higher-level, stylistic features such novelty, originality, or metaphoricity (Jacobs, 2015a). If this *null-model* of supralexical affective meaning were correct, a simple declarative sentence containing a positive noun and a negative adjective like “*The mother is bad*” should – on average – be evaluated as neutral. As counter-intuitive as this may sound, the seminal studies in *emotional stylometry* by Bestgen (1994) and Whissell (1994) both demonstrated that the valence of supralexical units (sentences, texts) could be predicted – to a considerable extent – as a function of the valence of their component words. Correlating the valence ratings for words and sentences taken from four different texts (***The Little Match Girl*** by Andersen, ***He Belonged to Me, Said the Sea*** by Cesbron, ***The Seven Ravens*** by the brothers Grimm, and ***The Stroll*** by de Maupassant), Bestgen (1994; Table 2) showed that word valence accounted for 30–60% of sentence valence, depending on the text. Even when considering potential effects of some methodological limitations regarding, e.g., the order of the ratings, these numbers are amazing.

Intrigued by the success of the null-model, Lüdtke and Jacobs (2015) tested the hypothesis that the supralexical affective structure is a linear combination of the valences of the nouns and adjectives contained in simple declarative sentences without any claim of being “beautiful” or “poetic.” Examples for most liked sentences in that study are “Die Kellnerin ist dankbar” (the waitress is grateful) or “Der Kuss ist romantisch” (the kiss is romantic); least liked were sentences such as “the waste is toxic”, or “Der Folterer ist gehässig” (the torturer is spiteful). The behavioral results obtained in an implicit task (sentence verification), that in contrast to Bestgen’s study did not require any attention to word valence and was context-free (i.e., no tale or story containing them), as well as in a second explicit rating task did not support the null-model. As concerns the latter task, the authors observed an interactive effect falsifying the linear combination model: sentences with positive and neutral adjectives after negative nouns (e.g., “Der Einbrecher ist schlau”/the burglar is smart) were evaluated more negatively than sentences with positive and neutral adjectives after positive nouns (e.g., “Die Geliebte ist sinnlich”/the lover is sensual).

This *negativity bias* for negative adjectives suggests an interaction of the general human negativity bias reported in the emotion processing literature discussed above and the syntactic role of words during sentence processing^4^. The authors interpreted this bias in terms of the above discussed asymmetry in semantic representations of negative vs. positive words: negative words are less homogenous, i.e., have less elaborated and cohesive representations (Ashby and Isen, 1999; Koch et al., 2016; Sylvester et al., 2016). Lüdtke and Jacobs (2015) therefore assumed that semantic integration and coherent situation model building for sentences with two negative words could be harder compared to sentences with two positive words. Since the condition in which the shortest verification times were observed, i.e., congruent positive nouns and adjectives, also received the highest valence ratings, Lüdtke and Jacobs also hypothesized that processing fluency and meaning construction are positively related to automatic valence evaluation, i.e., EADs. This supports the relation between perceptual and conceptual processing fluency and aesthetic pleasure, the link between memory associations and liking already discussed for single words, compounds, idioms, and proverbs. For sentences with two positive words, semantic activation can spread across better elaborated and connected associative pathways, and thereby elicit a positivity bias or *positivity superiority effect* during meaning construction.

In line with this, Havas et al. (2010) had already observed that reading times for what they called “happy” sentences like “You spring up the stairs to your lover’s apartment,” or “Finally, you reach the summit of the tall mountain” were significantly shorter than for sad sentences like “You open your email in-box on your birthday to find no new emails,” or “angry” sentences like “The workload from your pompous professor is unreasonable.” The authors tested and verified the prediction that paralysis of the corrugator supercilii (“frown muscle”) by Botulinum Toxin-A (botox) selectively hinders processing of angry and sad sentences, relative to happy sentences. Finding that BTX selectively slowed the reading of sentences that described situations that normally require the paralyzed muscle for expressing the emotions evoked by the sentences, they concluded that sentence comprehension involves a mental simulation of sentence content that calls on the same neural systems used in literal action, perception, and emotion.

Looking at more poetic materials than the simple declarative sentences, in a recent neuroimaging study, Citron and Goldberg (2014) found significantly enhanced activation of the left amygdala while participants read conventional, taste-related metaphors such as *She looked at him sweetly* compared with their literal counterparts, e.g., *She looked at him kindly.* Since the amygdala has been associated with automatic processing of highly emotionally arousing stimuli, as well as with salience or relevance detection, the authors suggested that metaphorical formulations *per se* may be more emotionally engaging than literal ones. This finding is in line with the results of two other studies already mentioned: Forgács et al’s. (2012) study on metaphorical and literal NNCs which also observed enhanced left amygdala activation for the former, and the meta-analysis of 23 neuroimaging studies on the comprehension of figurative statements (i.e., metaphors, idioms, proverbs, sarcastic, and ironic statements), in which activation of the left amygdala was reported for the contrast between figurative and literal statements (Bohrn et al., 2012a). Perhaps, given that amygdala activation is also associated with reward, if the amygdala is active in figurative language processing, it is being allowed to be so because the control regions of the prefrontal cortex-amygdala network linked to emotion regulation (e.g., Morawetz et al., 2016) detected positive affect. This might imply that metaphors and figurative language in general are, at least on an implicit neuronal level, more pleasant^5^.

To summarize, EADs to simple sentences can be related to enhanced meaning construction and processing dynamics based on the general principle of *good things come easy* (Unkelbach et al., 2010), which draws on Boucher and Osgood’s (1969) Pollyanna hypothesis (i.e., the universal human tendency to use positive words more frequently and diversely than negative ones in communicating) and on the mere exposure effect (Murphy and Zajonc, 1993). The structural argument in favor of a general processing advantage for positive stimuli, the informational density hypothesis^6^ (Unkelbach et al., 2008; Sylvester et al., 2016), is complemented by both a computational and phenomenological one: If positive stimuli are more densely clustered in memory, then people should experience higher subjective exposure to positive than to negative stimuli, because any positive stimulus has a greater likelihood of triggering thoughts of a large number of other associated positive stimuli (Hintzman, 1988; Hofmann and Jacobs, 2014). Thus, greater subjective exposure to (or familiarity with) positive words is an experiential consequence of the informational density hypothesis’ computational and structural arguments, as experimentally verified by Unkelbach et al. (2010).

In synopsis with what was concluded for words and multiword expressions, the liking of sentences thus seems to depend on higher subjective exposure and ease of processing for positive information. The participation of neural circuits for reward and relevance detection (medial orbitofrontal gyrus, caudate nucleus, amygdala) and, more generally, neural and bodily systems used in perception, action, and emotion fits with this interpretation, if we assume that under conditions of congruity they all contribute to the felt ease of processing and pleasure (Havas et al., 2010). From Kintsch’s (2012) computational perspective, the aesthetic liking or beauty of a nice line or sentence would be a complex function of harmony (i.e., minimal complexity), variety (i.e., distinctiveness of the parts), and compression (i.e., increasing harmony over processing time).

### Stories

As we move from smaller and simpler to longer and more complex text materials, the likelihood of identifying the key features that determine EADs, both on the text and the reader side, will hardly increase^7^. On the text side, one can analyze the potential structural key features via descriptive tools such as the 4 × 4 matrix that combines four text levels (metric, phonological, morpho-syntactic, semantic) with four groups of features (sublexical, lexical, interlexical, supralexical; Jacobs, 2015b). Many of these features can be quantified by appropriate tools like the BAWL, Coh-Metrix (Graesser et al., 2004) or TAACO (Crossley et al., 2015), and then fed into regression analyses to find out which features affect which response variables. On the reader side, longer pieces of text like stories and novels should increase the likelihood of triggering personal memories which play a key role for story comprehension (e.g., Larsen and Seilman, 1988; Pleh, 2003; Burke, 2011). It is beyond the scope of this paper to review even the most cited of the enormously broad and controversial literature on story processing (e.g., Wilensky, 1983, and commentaries to his paper), though. We therefore focus on a few aspects that seem most relevant for our computational modeling purposes (see Final section).

There are numerous papers on both the cognitive and emotional components of story processing (e.g., Brewer and Lichtenstein, 1982). In his review, Bestgen (1994) concludes that to adopt an integrated approach of interest and storyness, as advocated by Stein (1982), structural models of story grammar and cognitive interest (e.g., Golden and Rumelhart, 1993) should be complemented by methods of text analysis allowing the quantification of the emotional content in stories (cf. Miall, 1989). Using the four stories mentioned above, Bestgen could show that the more his readers liked the words and sentences of a story, the more they also liked the story itself (*R*^2^lin ranging from a minimum of 35% for the word-text valence correlation to a maximum of 70% for the sentence-text valence correlation for Maupassant’s story).

Good “storytelling, inevitably, is about compelling human plights that are “accessible” to readers.” (Bruner, 1986, p. 35), i.e., make the comprehension of the plans underlying the goal-directed actions of its protagonists easy by facilitating situation model building and mental simulation through theory of mind processes of empathy and identification which all facilitate immersion (e.g., Mason and Just, 2009; Mar, 2011; Altmann et al., 2012, 2014; Hsu et al., 2014; Jacobs and Lüdtke, in press). According to Brewer and Lichtenstein (1982) stories differ from narratives in that they are structured to evoke a particular affective response pattern in the readers. These authors identified three generic patterns, *suspense, surprise*, and *curiosity*, which correspond to different discourse structures and encode a distinct functional operation of the mind within stories’ overall intersequencing, i.e., the dynamics of *prospection, retrospection,* and *recognition*, respectively (Sternberg, 2003; cf. Jacobs, 2015b). Simply put in terms of Brewer and Lichtenstein’s theory, a story will be liked if it succeeds in arousing and resolving readers’ affective responses, e.g., suspense (Jose and Brewer, 1984; see also Berlyne, 1971; Lehne et al., 2015).

However, as has become clear from the preceding paragraphs, affective reader responses can be triggered at multiple levels of text by multiple features, from single words to the overall affective tone of the largest unit (e.g., a story). In addition, even though a story may have a dominant discourse structure of the type assumed by Brewer and Lichtenstein (1982), suspense, surprise, and curiosity responses can all happen in perhaps any kind of narrative thus making the theoretical liking function a more complex one than suggested by the former authors. Moreover, readers’ moods and motivations before choosing a text also are factors shaping their overall affective response, as are the socio-cultural frames and actual contexts of the story or book and the proper reading act (e.g., knowledge about authors, reading motivation, etc.; Burke, 2011; Jacobs, 2011).

Finally, while liking may be a compound but still relatively simple function of the affective reader responses in a certain type of literature, e.g., suspenseful crime stories, literary reading offers a wealth of other features that may trigger emotions and EADs. Thus, Miall (1989) was among the first to point out that defamiliarization or foregrounding (van Peer, 1986) also is an important source of affective reader responses and that readers of literary texts seek the experience of defamiliarization, i.e., look forward to redefine, modify, or suspend their schemata, and through this process explore the emotions of the self through engagement with the text. If Miall is correct, the latter process is the primary goal of reading.

At the neuronal level, the liking of (short) stories and text passages has been associated with bilateral medial prefrontal cortex, supramarginal gyrus/temporoparietal junction, and left dorsolateral prefrontal cortex (Altmann et al., 2012), the left posterior middle temporal gyrus (Hsu et al., 2015), and the medial frontal cortex (Lehne et al., 2015^8^).

### Poetry

Poetry can be very short, like Pound’s two-liner “In a Station of the Metro” or Quasimodo’s “And suddenly it’s evening” (Jacobs, 2016a), or, even more extreme, the one-word micropoetry described in Limbach’s (2004) book on the most beautiful German words of the year (Jacobs et al., 2015). It can also fill several pages like Schiller’s “Die Glocke” (The Song of the Bell, 430 lines) or a book like Homer’s Iliad. This is one reason why we deal with poems at the end of this section. Another reason is that poetry is often viewed as the highest or prototypical form of verbal art. A third reason is that poetry can generally be understood as inherently concerned with the expression and elicitation of emotions (Meyer-Sickendiek, 2011; Lüdtke et al., 2014) while being deeply rooted at the perceptual and aesthetic levels in the domains of speech and sound (Schrott and Jacobs, 2011).

Richards (1929) pioneered the study of poetry reception by having about 100 literature students read a set of 13 poems deemed of different quality and write down their impressions. The results of his method of open-ended responses – plus the lack of any statistical analysis – lead him to conclude that he got a hundred different verdicts from a hundred readers. In a statistical reanalysis of Richards’ data and two empirical studies using his poetic materials – one using ratings, the other essay writing, Martindale and Dailey (1995) could show that rater agreement was significant on 37/40 scales thus contradicting Richards’ often repeated subjective conclusion. On the liking scale, the agreement was very high, but inopportunely Martindale and Dailey (1995) provide no data specifying which of the 13 poems was liked most and why.

Trying to find quantitative structural predictors for the *aesthetic success* – a variable that is assumed to be highly correlated with liking – of the 154 Shakespeare sonnets, Simonton (1989, 1990) isolated four factors accounting for the superiority of very popular sonnets

(1) They treat *specific themes*, e.g., friendship, beauty, love, or death.
(2) They display considerable *thematic richness* in the number of issues discussed.
(3) They exhibit greater *linguistic complexity* as gaged by quantitative measures like the type-token ratio (i.e., the ratio of different words to total words) or adjective-verb quotient (i.e., the proportion of adjectives to verbs).
(4) They feature more *primary process imagery* (using the Regressive Imagery Dictionary/RID by Martindale, 1975).

Factor 1 was estimated by using the *Syntopicon* (Hutchins, 1952), a detailed topical index to the Great Books of the Western World. Overall, the 154 sonnets negotiate 24 different topics, some being variations on a key topic, such as intensity and power of love vs. altruistic love. Factor 2 basically is a count of the number of different topics within a sonnet^9^. Factor 3 reflects lexical variability and verbal complexity including variables like the number of unique words, an index of novelty. Finally, factor 4 is derived from words that associate primary process imagery in the Freudian sense, e.g., orality, sex, anality, etc.

In sum, according to Simonton’s computer-based analysis, the liking of Shakespeare sonnets can be related to a combination of the *specificity-variability*, *novelty-complexity*, and *primary process imagery* of the verbal material, all of which can be viewed to influence the arousal potential of a poem supposed to drive aesthetic pleasure (Berlyne, 1971; Cupchik, 1986). However, a recent more extensive and deeper computational-linguistic analysis of the 154 sonnets using a novel tool called *SPARSAR* challenges Simonton’s empirically not investigated deductions (Delmonte, 2016). Again, though, the computational data produced by SPARSAR remained empirically untested and will not be further discussed here.

Focusing on lexico-semantic textual properties, the pioneering works of Martindale and Simonton appear to neglect a key feature of poetry: Its sound structure and *euphony* (e.g., Tsur, 1992). A recent example for an attempt to predict the liking of entire poems by quantifying the euphony-eusemy nexus typical for poetry (i.e., *phonological iconicity*; Jakobson and Waugh, 1979/2002) is given in Aryani et al. (2016). The authors used the EMOPHON software (Aryani et al., 2013) to obtain quantitative estimates of the *basic affective tone* of poems from Enzensberger’s (1957/1981) collection “verteidigung der wölfe” (in defense of the wolves). The 20% of variance in valence ratings accounted for in the 57 poems by this measure strongly suggests that the iconic properties of foregrounded phonological units are a co-determinant of liking. While having shown effects of phonological iconicity on poetry reception is no news *per se*, the quantification of the distinctive features and the fact that on top of liking ratings the EMOPHON software also allowed to predict specific emotional responses, such as “spitefulness ratings” (*R*^2^lin = 0.22; see Table 2 in Aryani et al., 2016), makes the study a promising model for future approaches.

Ullrich et al. (in revision) offer an extended quantitative analysis of the reception of these 57 poems by Enzensberger, both on the text side and the reader response side. On the text side, based on the 4 × 4 matrix for textual analyses (Jacobs, 2015b), they added lexical and interlexical variables to the sublexical ones used in Aryani et al. to predict liking ratings. On the reader side, they added ratings for liking (in addition to valence ratings), poeticity, and onomatopoeia to those used by Aryani et al. For the present purposes, the most interesting results are: first, the strongest predictors of liking ratings were the valence and arousal values of the words in the poems, as computed using the BAWL (*R*^2^ = 0.47; *p* < 0.001). Although this is less variance accounted for than in the study by Bestgen (1994) using stories, it is worth noting that – at least in this extensive sample of German poetic texts – the affective-semantics of single words explain about half of the “liking cake.” Second, across all 57 poems, valence, and liking ratings were only moderately correlated (*r* = 0.59, *R*^2^ = 0.35; all *p* < 0.001). This value varied considerably for individual poems and poem categories, though. Thus, for sad poems, valence predicted liking better (*R*^2^ = 0.45; *p* < 0.0001) than for the two other groups: spiteful (*R*^2^ = 0.29; *p* < 0.025) and friendly (*R*^2^ = 0.23; *p* < 0.039). These variable, small to moderate, relationships between two reader response measures suggest that although ratings for valence, liking, or beauty seem to have some common latent factor (Lüdtke et al., 2014), they partially tap into different processes.

A third recent model-guided, multimethod study (Jacobs et al., 2016) used a mixture of tools for predicting aesthetic liking ratings, as well as other dependent variables, such as electrodermal activity, heart rate, or responses to the *Poetry Reception Questionnaire* for 24 German poems constituting the “mood poetry” corpus (Lüdtke et al., 2014). Similarly to Ullrich et al. (in revision), it quantified their distinctive features based on the 4 × 4 matrix and other text-analytic tools, but added a supralexical level: the motif group of the poems (Stillness, Space, City, and Morning). Here, the variance accounted for in liking ratings is of interest: supralexical factor motif (*R*^2^ = 0.39, *p* < 0.017), interlexical factors valence span, and arousal span (*R*^2^ = 0.14, *p* < 0.018 and 0.13, *p* < 0.019, respectively). Furthermore, one sublexical (phonological iconicity) and 10 lexical variables significantly affected liking ratings (**Table 1**), showing a degree of complexity comparable to that of the single word VDT study by Jacobs et al. (2015).

**Table 1:** Stimulus features and neural networks relevant for liking verbal materials.

Verbal stimulus	Relevant features	Relevant neural networks^1^
Words	valence, arousal, imageability, frequency/familiarity, number of syllables, neighborhood density, joy/happiness, fear, anger, sadness, disgust, taste, grasp, move	orbitofrontal and insular cortex, middle temporal and left inferior frontal gyrus, hippocampus
Idioms	familiarity, arousal, figurativeness	left pre- and post-central gyri, right superior temporal gyrus
(Anti-)Proverbs	familiarity, rhyme, meter, prägnanz	medial orbitofrontal cortex, striatum
Sentences	valence congruity, figurativeness, prägnanz, harmony, variety, compression	left amygdala, medial orbitofrontal gyrus, caudate nucleus
Stories	valence, arousal, features evoking suspense, surprise and curiosity	bilateral medial prefrontal cortex, supramarginal gyrus/temporoparietal junction, left dorsolateral prefrontal cortex, left posterior middle temporal gyrus
Poems	basic affective tone (phonological iconicity), word valence and arousal, imageability, taste, grasp, move, joy, fear, anger, sadness, disgust, valence and arousal span, motif, specificity, thematic richness, linguistic complexity, primary process imagery	bilateral precentral and inferior frontal gyrus, right dorsolateral prefrontal gyrus, anterior insula, temporal pole, posterior/mid-cingulate, parahippocampal and left superior temporal gyrus, bilateral hippocampus

*^1^Only networks specifically related to (dis-)liking are mentioned excluding all other structures related to, e.g., visual-auditory language processing. The exception are poems for which the cited studies provided no data about liking; here the relevant networks refer to those that are recruited during poetry vs. prose reading.*

Neuronal correlates of processing (not of liking) poetic (vs. non-poetic) texts are the bilateral precentral and inferior frontal gyrus, as well as the right dorsolateral prefrontal cortex extending into the anterior insula, and beyond to the temporal pole. Interestingly, the dorsomedial prefrontal cortex showed reduced activation during reading of poetic pieces, compared to the reading of prosaic pieces (O’Sullivan et al., 2015). Further areas specifically related to poetry reception are the right posterior/mid-cingulate, parahippocampal, and left superior temporal gyrus, as well as bilateral hippocampus (Zeman et al., 2013).

To summarize, as hypothesized by the NCPM, liking decisions concerning poetry are affected by a multitude of sublexical, lexical, interlexical, and supralexical factors at all four text levels considered by Jakobson (metric, phonological, morpho-syntactic, and semantic). Their dynamic interactions make it difficult to obtain a clear picture about the relative weight each factor has in determining EADs. Without proper process models (e.g., Leder et al., 2004, for visual arts; Jacobs, 2011, 2015a; for literary reading) or computational models (e.g., Hofmann and Jacobs, 2014, for word recognition) offering testable predictions at complementing levels of observation (i.e., neuronal, experiential, behavioral), the hidden structure and dynamics underlying EADs and aesthetic liking for complex verbal materials will be hard to uncover.

## Summary of Previous Sections

To facilitate integration of the previous sections, **Table 1** summarizes, in a very simplified way, the key pieces of information relevant for EADs to verbal materials of increasing complexity. The data in the various cells of this Table are hard to compare or integrate into a meaningful picture, though, because the materials, tasks, and methods used in the studies producing them were simply too heterogeneous. What would be needed for an integrative account of EADs to different verbal materials are studies comparing, say, literal and figurative sentences, story passages or poems where all materials are analyzed with the same text-analytical tools (to identify quantitative predictors) and in which the same task and method is used (e.g., liking ratings, VDT, fMRI). Meanwhile, **Table 1** can serve as a first orienting tool for future studies in Neurocognitive Poetics and affective decision making informing researchers about which factors they should carefully consider using as independent or controlled/matched variables in their designs. An important task for future studies is to determine the relative weight and potential dynamic interactions of these features. Moreover, column three of **Table 1** could help to select regions of interest or seed regions for neuroimaging studies using psychophysiological interaction (PPI) or dynamic causal modeling (DCM) analyses (e.g., Altmann et al., 2012; Lehne et al., 2015; Pehrs et al., 2015).

## Decision Tree (Recursive Partitioning) Modeling of Eads to Single Words

As stated above, the number of factors involved in determining EADs to longer and more complex text materials such as stories, poems, or novels represents a huge challenge to any modeling approach. First descriptive models have taken up this challenge only recently, e.g., the NCPM (Jacobs, 2011, 2015a, 2016b) or Burke’s (2011) model of reading novels, but the way to formal computational models has yet to be paved by further research sufficiently constraining theory development (for a review of model types, see Jacobs and Grainger, 1994 or Hofmann and Jacobs, 2014). Note that even EADs to simple verbal materials such as single words still have not been modeled computationally, despite a wealth of formal models in the field of decision theory and neuroeconomics (e.g., Rangel and Clithero, 2014). One reason is that performance in the VDT, i.e., RTs reflecting positive vs. negative decisions, may depend on more than a dozen quantifiable factors. To prepare development of more formal modeling tools, we recently have proposed three descriptive neurocognitive models of the VDT (Jacobs et al., 2015).

In this section, we go a step further by adopting an exemplary, formal decision tree modeling approach – a standard data mining/machine learning technique – to illustrate how EADs to high-dimensional stimuli such as words (or text segments) can be predicted. A potent use of decision tree modeling is exploring relationships without having a good prior theoretical model: It can handle even large data problems efficiently allowing to test clear hypotheses, and the results are usually transparent and easily interpretable^10^. Here we were interested in asking the question which of 10 quantitative features of words related to their affective-semantics and empirically established as potentially relevant factors was most important in determining EADs.

## Stepwise Modeling Approach

Similar to Jacobs et al’s. (2015) statistical analyses, we used a stepwise approach going from simple to complex models to see how much complexity in the input space is necessary to obtain an adequate model performance. All models were trained on 70% of the words (the randomly chosen training set is the part that estimates model parameters) and then validated on the remaining 30% (the validation set is the part that addresses or validates the predictive ability of the model). The stimuli were the *N* = 91 (46 negative and 45 positive) words used in Jacobs et al’s. (2015) reanalysis of the original BAWL study for which a large number of quantifiable features are available. We started with a simple two-variable “affective-semantic” model that implemented the hypothesis that EADs to words depend on their arousal and imageability values, as quantified in the BAWL database (cf. Jacobs et al., 2015). The more complicated three-variable “embodiment” model tests the hypothesis that EADs mainly depend on word features related to embodied associations (taste, grasp, move). The next five-variable model posits that the associations with five discrete “basic” emotions (Joy, Fear, Anger, Sadness, Disgust) determine EADs. Finally the most complex model included all 10 variables of the previous models. The models were implemented using the PARTITION tool of the JMP Pro 11 software and model performance was gaged by the number of correct EADs, i.e., whether the model classified a target word correctly as either positive or negative (corresponding to the norm values of the BAWL database). Descriptively, model performance is expressed by the number of partitions, i.e., how many decisions are required to obtain maximum accuracy, *R*^2^ and the number of misclassifications, i.e., how often the model classified a positive stimulus as negative or vice versa.

**Table 2** summarizes the results. Each model in the table implements and tests a different hypothesis concerning the factors determining EADs to single words, e.g., Model 1 tests to what extent EADs are driven by two affective-semantic features only.

**Table 2:** Input variables and performance evaluation for four decision tree models of the valence decision task (VDT) with single words.

Model	Input variables	Model performance (Nbr of partitions, *R*^2^, misclassifications)
(1) Affective-semantic	Arousal, imageability	6,.79, 6
(2) Embodiment	Taste, grasp, move	12,.43, 18
(3) Discrete	Joy, fear, anger, sadness, disgust	2,.94, 1
(4) All 10 features	All above features	2,.94, 1

Descriptively, models 3 and 4 are the winners of this competition both producing an almost perfect performance with only one misclassification, i.e., 99% correct responses in valence decisions to single words. Thus, somewhat surprisingly, the data in **Table 2** suggest that the most complex model with 10 input features is not necessarily the best predictor of EADs, since the simpler five-variable discrete emotion model fared as well.

**FIGURE 1:**
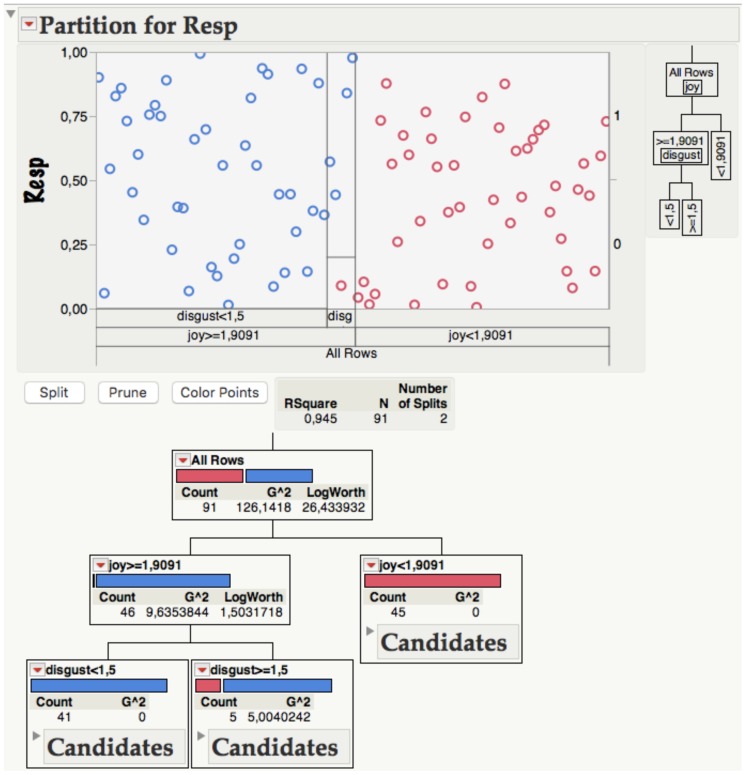
**Upper panel**. Partitioning results and decision tree report for 46 negative (red) and 45 positive (blue) words as implemented in Model 3. Resp, response (positive vs. negative; see text for details). **Lower panel**. Detailed decision tree for Model 3 with number of candidates, *G*^2^ values indicating the likelihood ratio χ^2^ for the best split, and LogWorth statistics [defined as – log10(*p*-value)]. The optimal split is the one that maximizes the LogWorth.

Can we tentatively infer that EADs to words are determined by their associations to five basic emotions then (Westbury et al., 2014)? **Figure 1** illustrating the decision tree data for Model 3 suggests an even simpler answer: to obtain 99% correct EADs, the model requires only two questions. Question 1 is whether the JOY/HAPPINESS value of a word is bigger than or equal to 1.9 (on the five point scale of the BAWL). If the answer is “No” the EAD will be “Negative” (0 in this case; red circles). Question 2 then applies to words for which the answer is “Yes” (1; blue circles) and is whether their DISGUST value is smaller than 1.5. If it is, the EAD will be positive, i.e., the word will get a “thumb up” or LIKE.

The results illustrated in **Figure 1** tempt a straightforward interpretation: what basically determines EADs to words from our empirically well cross-validated BAWL sample is the degree to which they are associated with joy or happiness. To a lesser extent, disgust associations also play a role. Associations with the other three discrete emotions (sadness, anger, fear), however, are not really useful for EADs in this context. That joy apparently plays a much stronger role in EADs as simulated by our Model 3 fits well with the positivity superiority effect mentioned above (Lüdtke and Jacobs, 2015) which can be interpreted by the hypothesis that the hippocampus is more generally involved in the processing of positive affect (Hofmann and Kuchinke, 2015).

Given the limitations of this heuristic modeling approach (a rather small sample of 91 German stimuli; only single words), any interpretation can only be tentative, though. Nevertheless, these results fit well with the evolutionary and appraisal accounts of aesthetic emotions discussed in the “Introduction” and also with data from recent neurocognitive studies using either lexical or VDTs (Briesemeister et al., 2014, 2015; Kuhlmann et al., 2016) and thus are further (computational) evidence for our specific hypothesis stated in the “Introduction.” Together these results suggest that valence is indeed a compound *superfeature* neuronally computed at the so-called tertiary (i.e., neocortical) level of affective processing according to Panksepp’s (1998) hierarchical theory of emotions. In contrast, joy/happiness and disgust are more basic and central affective responses likely computed at the secondary level (i.e., the limbic system). The neuroimaging results from Briesemeister et al. (2015) indicate that words associated with joy produce reduced brain activity in the amygdala, i.e., at the secondary level of Panksepp’s theory, while words that have positive valence, but are not associated with the basic emotion joy/happiness activate the orbitofrontal cortex at the tertiary level of affective processing. Further evidence for this comes from a brain-electrical experiment using the same stimuli and indicating that joy-words affect the early N1 component of the ERP – known to be sensitive to affective conditioning (Fritsch and Kuchinke, 2013) –, while positively valenced words affect the later N400 and LPC responses. Additional evidence stems from the above mentioned study by Ponz et al. (2013) showing that the anterior insula – which is also considered part of the secondary level – is activated during the reading of disgusting words. The authors interpreted this finding in terms of neural reuse and the Panksepp-Jakobson hypothesis (see “Introduction”) suggesting that phylogenetically younger processes such as reading rely at least partially on already-existing ancient affective circuits like the limbic system. Words strongly associated with a given emotion are thus assumed to activate the corresponding conditioned affect program more strongly than words judged as being weakly related to that emotion, or than neutral words.

To summarize, the results from our decision tree modeling together with those from recent neurocognitive studies allow to simplify our initial main hypothesis for straightforward testing in future studies on EADs and aesthetic liking: The extent to which high-dimensional stimuli such as words are associated with two basic emotions – likely to be evoked at the secondary level in Panksepp’s (1998) emotion theory –, namely joy/happiness and disgust, drives EADs in a considerable and quantitatively predictable way. Whether this is the case only for single words (for which the necessary feature values can be found in databases like the BAWL) or whether it can be generalized to more complex verbal and non-verbal materials is an open issue for future studies. Note that according to this simplified hypothesis the role played by associations to other basic emotions (e.g., fear, sadness, anger) or by other affective and embodied features (e.g., arousal, taste) seems to be a relatively minor one. The empirical data of the study by Jacobs et al. (2016) would suggest that with complex materials such as poems also associations with these other features gain in weight, but implementing the 24 poems studied by these authors in a decision tree or other formal model clearly is beyond the scope of this paper and will thus await future research.

## Conclusion and Outlook

We started out with Virginia Woolf’s question *How did one add up this and that and conclude that it is liking one felt, or disliking?* Based on previous empirical and theoretical work from our group, we submitted the hypothesis that the main factor used in the investigation of EADs, i.e., stimulus valence, is a semantic *superfeature* resulting from a yet unknown integration of experiential and distributional data, at least partially represented in associative activation patterns of affective-semantic networks starting out in parts of the limbic system (Ponz et al., 2013; Briesemeister et al., 2014, 2015; Jacobs et al., 2015; Kuhlmann et al., 2016).

Our review of methods and materials used in the scientific study of EADs, as well as of the factors determining the liking of verbal materials with increasing degrees of complexity provided accumulated empirical evidence for this assumption. A final section presenting formal decision tree models of EADs with different degrees of complexity (2–10 input variables) also supported the hypothesis and suggested a most parsimonious version, i.e., a compression to only two relevant, unevenly weighted dimensions of the semantic space supporting EADs to single words: joy/happiness and disgust.

Thus, to use Woolf’s words, what is *added up* for concluding a LIKE or DISLIKE decision, at the neuronal level probably relates to two ancient core affect programs, well described in Panksepp’s (1998) emotion theory. Liking and beauty ratings may be a complex context-dependent function of many variables, and so may be EADs (Güçlütürk et al., 2016; Marin et al., 2016), but perhaps in the end it always involves implicit associations with joyful or disgusting events triggered by the stimulus, the former taking a greater weight in the decision. Future studies on EADs should thus pay more attention to the underlying (asymmetric) affective-semantic structure (i.e., relating to discrete or embodied emotions) of the stimuli used, before a priori polarizing them symmetrically into “positive” or “negative” ones. Studies in neuroeconomics focusing on the reward values of stimuli and actions (e.g., Rangel and Clithero, 2014) should pay more attention to the idea that although EADs entail no direct overt reward, they may well be guided by unevenly weighted *intrinsic rewards* based on remembrance of things past.

This idea, of course, is not new. For example, Epstein’s (2004) neuroaesthetics theory, inspired by Proust and James, claims that the function of art is to evoke the underlying associative network indirectly in the mind of the observer by using carefully chosen sensory surfaces to control the stream of thought and induce pleasure and aesthetic liking. This involves distinct neural/cognitive mechanisms, including a network of associations supported largely by the medial temporal lobes (e.g., hippocampus) that determines the relationship between the current *nucleus* and other potential thoughts and feelings forming the *fringe* (for a more detailed treatment, cf. Jacobs, 2015b). The challenge is to uncover these mechanisms in all their apparent complexity by combining neurocognitive studies and computational modeling, as tentatively exemplified in this paper.

A final word, lent from Kintsch (2012, p. 646), on the liking of verbal stimuli that were left out from the present paper is in order: books. “Why is it that we can read a great book many times and it becomes more interesting with each reading? Because it affords us the opportunity to fine-tune our (mental) model, to construct a novel interpretation every time. The book remains the same, but we – our model – change.”

## Author Contributions

AJ: Paper conception and major writing. MH and AK: minor writing.

## Conflict of Interest Statement

The authors declare that the research was conducted in the absence of any commercial or financial relationships that could be construed as a potential conflict of interest.

## Footnotes

1 Note that aesthetic processing is best thought of as a binary phenomenon, with both positive and negative counterparts. Thus, negative-valenced emotions such as dislike and disgust are just as much aesthetic emotions as are awe and ecstasy (Brown et al., 2011).

2 Synonyms used in the literature are valence identification or classification, valence judgement, affective decision, or aesthetic judgement task.

3 Valence was found to be the strongest predictor for beauty ratings (*R*^2^lin = 0.77).

4 The higher arousal values for both negative adjectives and nouns than for positive ones likely played a role here. Since Bestgen (1994) did not measure/report arousal values, we can only speculate that apart from the lack of context, this is another potential source for discrepant results.

5 The authors thank review editor Matthew Pelowski for this interesting suggestion.

6 Positive information is more similar and is more closely related to other positive information than negative information is to other negative information, thus affording less precise mental representations and decisions/actions than negative information.

7 Here, we exclude micro-narratives like Hemingway’s famous “*For Sale: Baby Shoes, Never Worn”* which is based on what is left unsaid.

8 The latter result is indirect in the sense that Lehne et al.’s parametric effects relate to suspense ratings which correlated very highly with liking (reading pleasure) ratings (*r* = 0.89), as argued, e.g., by Jose and Brewer (1984).

9 Generally, more highly esteemed products tend to treat a broader range of themes (Simonton, 1983).

10 A classic application is when you want to turn a data table of symptoms and diagnoses of a certain illness into a hierarchy of questions. These questions help diagnose new patients more quickly.
